# Yolk–Shell Nanostructures: Syntheses and Applications for Lithium-Ion Battery Anodes

**DOI:** 10.3390/nano10040675

**Published:** 2020-04-03

**Authors:** Geon Dae Moon

**Affiliations:** Dongnam Regional Division, Korea Institute of Industrial Technology, Busan 46938, Korea; gmoon@kitech.re.kr; Tel.: +82-55-912-0333

**Keywords:** yolk–shell, templating, self-templating, battery anode, nanomaterial

## Abstract

Yolk–shell nanostructures have attracted tremendous research interest due to their physicochemical properties and unique morphological features stemming from a movable core within a hollow shell. The structural potential for tuning inner space is the focal point of the yolk–shell nanostructures in a way that they can solve the long-lasted problem such as volume expansion and deterioration of lithium-ion battery electrodes. This review gives a comprehensive overview of the design, synthesis, and battery anode applications of yolk–shell nanostructures. The synthetic strategies for yolk–shell nanostructures consist of two categories: templating and self-templating methods. While the templating approach is straightforward in a way that the inner void is formed by removing the sacrificial layer, the self-templating methods cover various different strategies including galvanic replacement, Kirkendall effect, Ostwald ripening, partial removal of core, core injection, core contraction, and surface-protected etching. The battery anode applications of yolk–shell nanostructures are discussed by dividing into alloying and conversion types with details on the synthetic strategies. A successful design of yolk–shell nanostructures battery anodes achieved the improved reversible capacity compared to their bare morphologies (e.g., no capacity retention in 300 cycles for Si@C yolk–shell vs. capacity fading in 10 cycles for Si@C core–shell). This review ends with a summary and concluding remark yolk–shell nanostructures.

## 1. Introduction

Energy storage from renewable energy production to electrical energy upgrades the status of lithium-ion batteries to a more significant position due to its large capacity, long lifespan, and high energy density [1]. Among the configuration of rechargeable batteries, the anode is an important part in lithium/sodium-ion batteries in terms of the following requirements: 1) high specific surface area for higher lithium/sodium ion insertion channels, 2) low volume change during charge/discharge for good cycling stability and safety, 3) large pore size and short path for high rate capability, 4) low internal resistance for fast charging, 5) low intercalation potential for Li or Na, 6) price competitiveness, and 7) environmentally-friendliness [2,3,4]. Since the commercial use of graphite as anode material in LIB, its low gravimetric capacity (graphite, 372 mAh/g) provoked the exponentially-increasing R&D needs for high capacity anode materials to meet the requirement of high energy density lithium-ion batteries for electrical vehicles, smart grid systems, and aerospace applications. An urgency to replace the conventional graphite anodes focused research efforts into lithium metal with higher energy density. Even though lithium metal has one of the highest capacities and the lowest potential, safety issues keep the use of lithium as anode material in LIB due to dendrite formation on lithium metal leading to short-circuit. Thus, alternative candidates have been emerging as new anode materials to tackle the safety problem. Based on the way of Li-ion storage, anode materials are classified into alloying anodes (Si, Sn, Ge, Al, SnO_2_), intercalating anodes (carbon, TiO_2,_ LTO), and conversion reaction anodes (transition metal oxides, chalcogenides, phosphides, nitrides). 

Compared to bulk silicon anode, nanoscale silicon materials have been demonstrated as an effective strategy since nanostructured Si can accommodate elevated mechanical stress leading to prolonged cycling stability. Nevertheless, such nanostructured Si anodes still suffer from short cycle life due to the loss of active material and increased cell impedance at high mass loading [5,6]. Other alloy-based anode materials, such as Sn and Ge, have long been considered as alternatives due to their high specific capacities and lower operating potentials than graphite. In addition, tin has low electrical resistivity (1.1 × 10^−7^ Ω·m). Tin and germanium are also prone to severe capacity degradation and short cycle life, caused by their large volume changes during lithiation/delithiation, subsequent pulverization, the formation of SEI, inhibition of Li^+^/electron transport, and delamination from current collector [7,8]. Metal oxides are also a promising family of anode materials since they have a safer lithiation potential that eliminates the problematic lithium dendrite formation during charging. However, the low electrical conductivity of metal oxides demands the conductive material in anode structures. Nanostructured metal oxides and their composites with various carbon materials have been used to improve their capacity retention by suppressing phase segregation, volumetric expansion, and ionic/electronic transport [9]. There have been recent research efforts to develop other anode materials including metal chalcogenides, phosphides, and nitrides. Like other transitional metal oxides, these metal compound anodes are based on the conversion reaction. Although these metal compounds have higher operating potential than graphite, they are still attractive candidates due to higher theoretical capacities [2,10,11,12,13,14]. 

Unlike the intercalating-type anodes with ignorable volume expansion, the chemical reaction between the anode with lithium ions accompanies serious volume change leading to an increase of internal resistance and rapid capacity decay. Due to the large volume change during charging/discharging in the anode, there needs to protect active material from fracture or dendrite formation. A number of structures have also been investigated in order to boost the capacity of lithium/sodium-ion batteries [15,16]. As one of the successful ways, researchers have paid attention on designing yolk–shell nanostructure to place active anode materials in hollow protecting layers [17,18,19,20]. Various research has already been done on the yolk–shell nanostructures for applications of drug delivery, sensor, and catalyst [21,22,23,24]. Different from core–shell structure in dense contact, yolk–shell nanostructures create movable space inside the protecting shell, which enables anode to expand without fragmentation or dendrite formation during a chemical reaction. Thus, yolk–shell nanostructures are beneficial for improving electrochemical performance owing to various merits including buffering space, large surface area, and short diffusion path. 

In this review, we will provide a recent progress of battery anode materials based on yolk–shell nanostructures. For the starter, we give a general explanation on the development of how to build up yolk–shell nanostructures through templating and self-templating approaches. Then, we move on to discuss various yolk–shell nanostructures for application in lithium-ion battery anodes. Lastly, the concluding remark follows in the conclusion and outlook.

## 2. Building Yolk–Shell Nanostructure

**Scheme 1:**
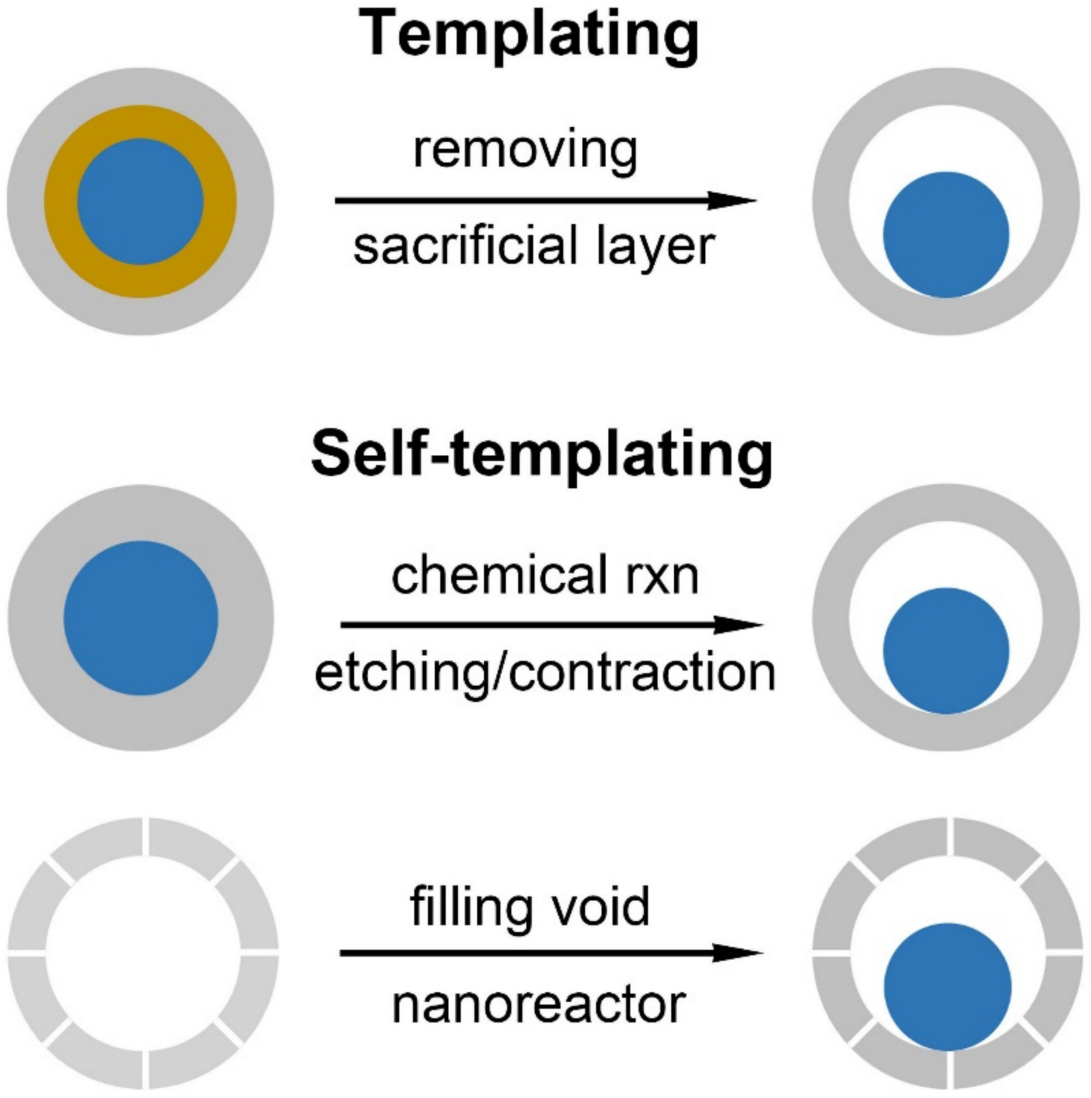
Synthetic strategies towards yolk–shell nanostructures categorizing into the templating and the self-templating approaches.

Yolk–shell nanostructures indicate a class of hybrid materials consisting of a hollow shell encircling movable cores with the void. The hollow nanostructures containing different nanoparticles inside possess unique morphological features, which bring low density, large surface area, and great loading capacity. In addition, the yolk–shell nanostructures can create hybrid materials with complex functions for specific purposes by integrating various functional components into this system. Thus, a lot of attention has been paid to this promising structure since it has great potential for diverse applications in catalysis, nanoreactors, drug delivery, and energy storage [25,26,27,28,29,30]. The existence of the void inside allows the exposure of the inner materials with a protective effect of the shell, which is advantageous in the preservation of the core materials. The void space in the yolk–shell nanostructures can serve as a storage space for functional cargoes and as a reaction room for chemical reactions. Compared to the conventional core–shell structures, the yolk–shell nanostructures offer additional availability for the tuning of materials. As is depicted in the Scheme 1, the yolk–shell morphologies can be achieved by straightforwardly templating methods as well as self-templating syntheses without additional templates for the formation of the cavity.

### 2.1. Templating Methods

**Figure 1:**
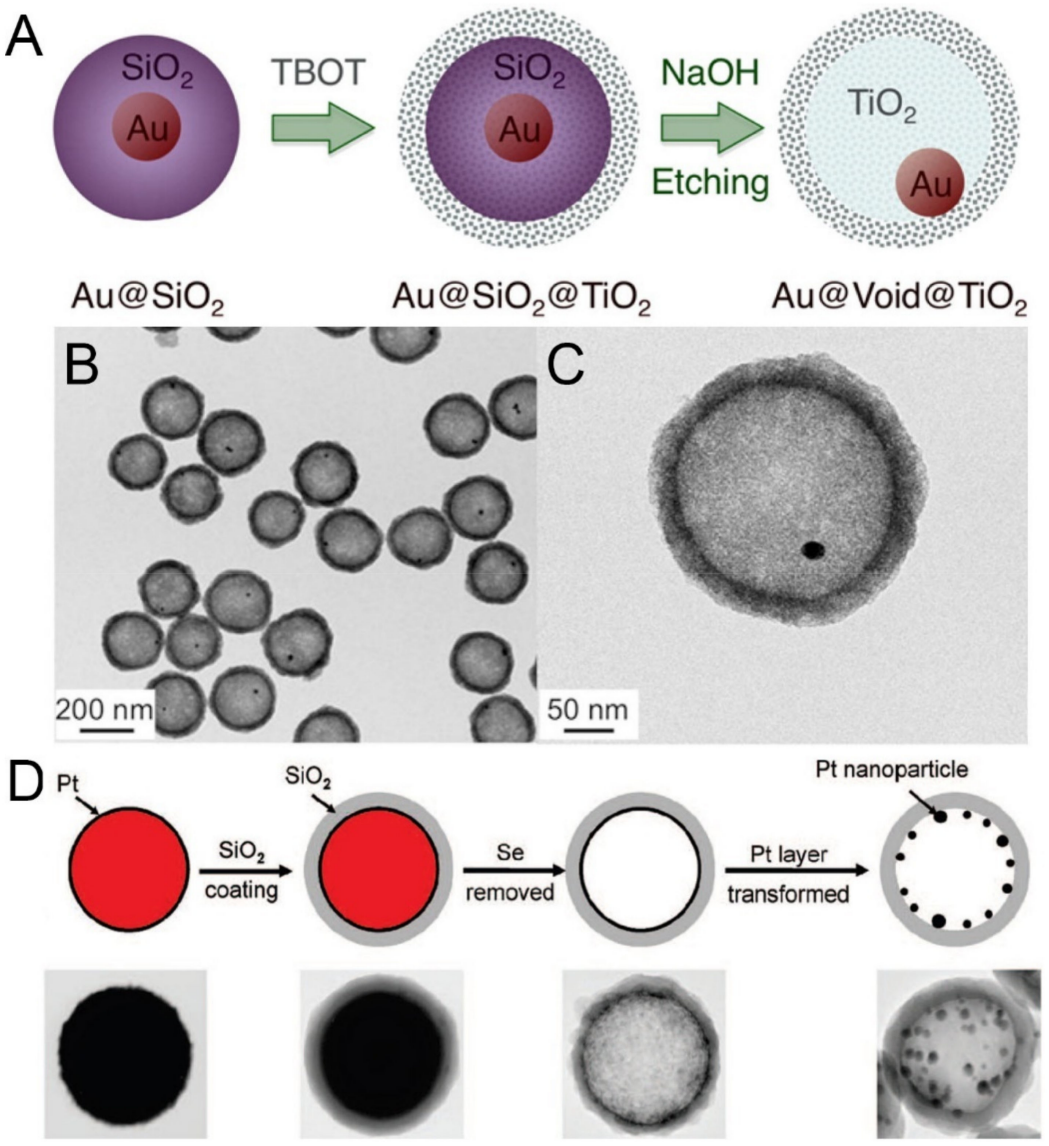
Templating approaches for yolk–shell nanostructures. (**A**) Synthetic scheme of Au@TiO_2_ yolk–shell nanostructure by using SiO_2_ as a sacrificial layer. (**B**,**C**) TEM images of the Au@TiO_2_ yolk–shell after etching the SiO_2_ layer. Reproduced with permission from [31]. Copyright Elsevier Ltd, 2016. (**D**) Decoration of interior surfaces in the SiO_2_ hollow shell by exploiting amorphous Se colloids as a template. Reproduced with permission from [35]. Copyright American Chemical Society, 2008.

Templating method is the most common approach for building up yolk–shell nanostructures because it is conceptually straightforward in a way that templates can be removed leaving the cavity inside the shell. Templating synthesis is classified into hard-templating and soft-templating based on the kind of template materials, i.e., rigid or soft. Generally, templating approaches follow the steps below: formation of the core, template coating, coating of the outer layer, and removal of the sacrificial template. In some cases, the surface modification is necessary for the coating of the outer layer to confer suitable surface properties. The removal of the template can be done through chemical etching, solvent dissolution, and calcination to obtain the cavity inside the hollow shell. Figure 1A shows the synthetic scheme of yolk–shell nanostructured Au@TiO_2_ morphology for the cryogenic oxidation reaction catalyst of carbon monoxide [31]. Gold nanoparticles acting as the yolk were first coated with silica to form Au@SiO_2_ core–shell nanostructure through a sol–gel process. Then, an outer titania shell was deposited on the surface of silica using tetrabutyl titanate (TBOT) to generate Au@SiO_2_@TiO_2_ double-shell nanostructure. Finally, the silica was etched away by using an alkaline solution to create the void with the Au@TiO_2_ yolk–shell nanostructure. The resultant image of Au@TiO_2_ yolk–shell nanostructures is displayed in the TEM images of Figure 1B,C. The use of Au nanoparticles in catalyst has one limitation, which they are easy to sinter due to the size effect. This leads to the severe degradation of catalytic activity. Thus, the authors adopted the yolk–shell architecture as an appropriate nanoreactor for the catalytic reaction since the titania shell can isolate the gold nanoparticles from clustering and guarantee the transfer of gases in and out of the inner space for sufficient access to gold surfaces. Typical materials for templating are silica, carbon, metal, metal oxides, and oligomers [32,33]. Silica is one of the most frequently used hard templates for the fabrication of yolk–shell nanostructures due to its easy removal with alkali or hydrogen fluoride treatment. For example, Shi and co-workers reported that Au@mSiO_2_ and ellipsoidal Fe_2_O_3_@mSiO_2_ yolk–shell nanostructures by using sodium carbonate and ammonia solution as the etching agent [34]. Carbon has also been used as a hard template due to its easy removal through calcination. Spindle Fe_2_O_3_@mSiO_2_ yolk–shell nanostructures were reported by using carbon as the sacrificial template [23]. In general, the firstly-coated shell act as a sacrificial template to generate voids inside the hollow shells after the removal process. However, the innermost core material can also behave as the removal template for the formation of desired materials inside hollow shells. Figure 1D exhibits the schematic procedure of fabricating platinum nanoparticle-decorated hollow silica shell [35]. The platinum layer was coated on the surface of amorphous selenium colloids through chemical reduction in alcohol. Then, the Stöber process produces the silica coating on the surface of Pt shell to finally form Se@Pt@SiO_2_ double-shelled structures. The innermost core was removed by dissolving in appropriate solvent like alcohol and hydrazine. During the removal process, the Pt layer go through transformation into Pt nanoparticles depending on the material flux. While the slow dissolution of Se core tends to preserve the thin Pt layer, the high flux of the core induces the rearrangement of the Pt clusters into nanoparticles ranging from a few to tens of nanometers by adjusting the temperature and the solubility of the Se core in solvents. 

### 2.2. Self-Templating Methods

**Figure 2:**
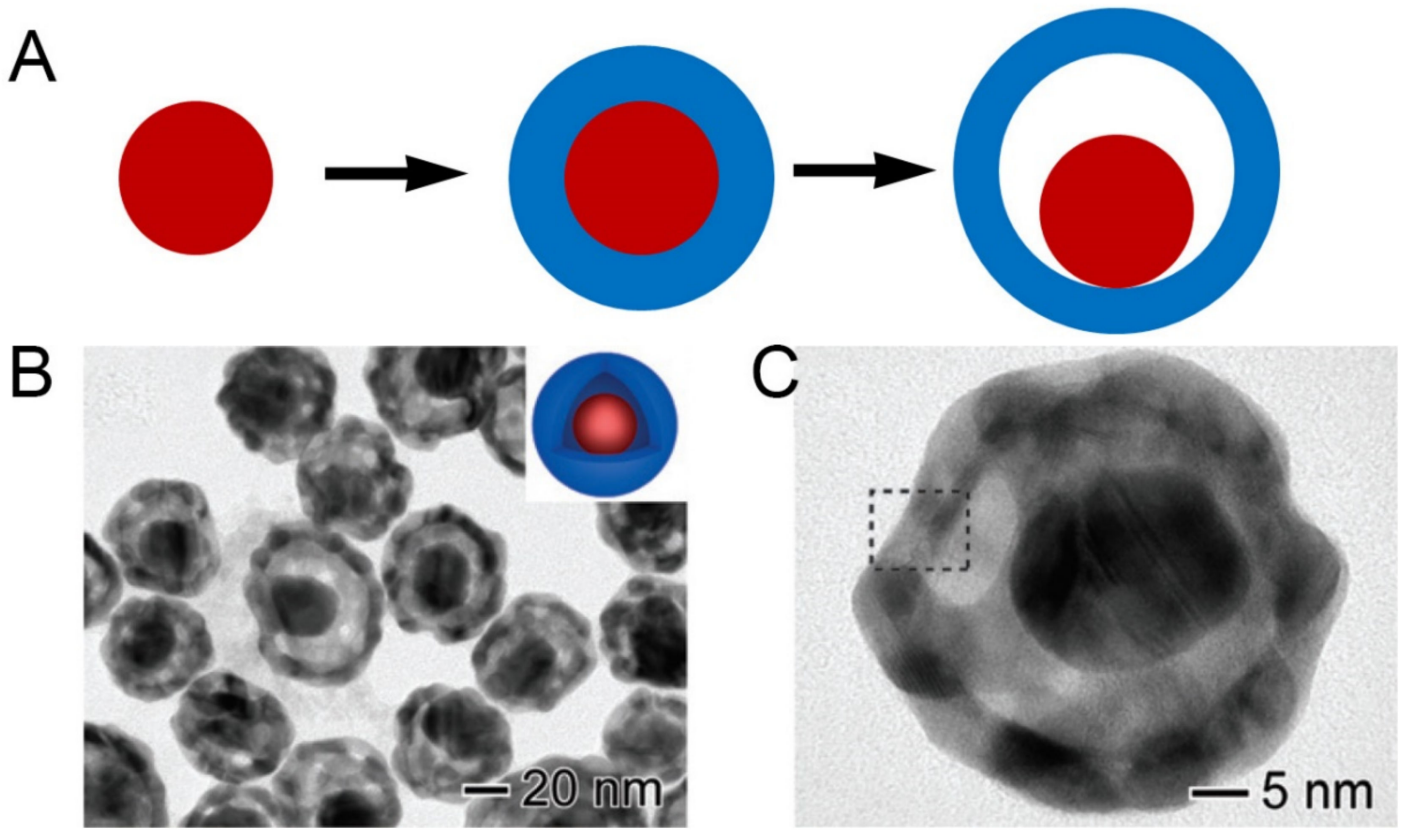
Self-templating syntheses for yolk–shell nanostructures. (**A**) Scheme illustrating the procedure for the Au core and Au/Ag alloy shell through the galvanic replacement reaction. (**B**,**C**) TEM images of Au@Au/Ag yolk–shell nanostructure. Reproduced with permission from [37]. Copyright Wiley-VCH, 2016.

Unlike the conventional templating methods, direct syntheses of yolk–shell nanostructures have also been developed by self-templating approaches. The self-templating methods can contain the following categories: galvanic replacement, Kirkendall effect, Ostwald ripening, partial removal of the core, core injection, core contraction, and surface-protected etching. The galvanic replacement reaction happens at the interface of metals with the different electrical potential, in which one metal serves as a reducing agent and the other metal serves as an oxidizing agent, respectively. As a result, the metal with low reduction potential tends to oxidize by reducing the other metal ion into a reduced form. Generally, the core metal is coated with other metal which has higher reduction potential. Upon addition of the metal ions, the core metal tends to dissolve in the solution while the metal ions are reduced and plated on the surface of the core metal. Xia and co-workers reported the synthesis of Au/Ag@Au/Ag yolk–shell nanostructures based on the reduction potential difference between Au and Ag [36]. Figure 2 shows the illustration of the process for fabricating Au@Au/Ag yolk–shell nanostructure starting from Au@Ag core–shell through the galvanic replacement reaction [37]. During the reaction, the Ag shell is transformed into Au/Ag alloy by creating the hollow void between the core and the shell. The Kirkendall effect is also an efficient way to prepare yolk–shell nanostructures since the motion of the interface between two materials occurs due to different diffusion rates of atoms or ions. Alivisatos and co-workers reported that Au@iron oxide yolk–shell nanoparticles can be synthesized through the Kirkendall effect [38]. This process starts with the deposition of an iron shell on an Au core, followed by the oxidation of the iron shell into a hollow iron oxide shell as well as void formation inside. Ostwald ripening is also a well-known reaction that involves the dissolution of small crystals and the redeposition of the dissolved species on the surfaces of larger crystals. Recently, Caruso and co-workers demonstrated that titania@ammonium titanate yolk–shell nanostructures can be synthesized by the self-templating approach based on the Ostwald ripening mechanism [39]. A little more straightforward self-templating method is the partial removal of the core from core–shell structures. Core–shell structures with a core material being able to etched or dissolved can create void space inside the shell of the core–shell morphology. For example, Paik and co-workers reported the synthesis of Fe_3_O_4_@C yolk–shell nanocubes through the partial removal approach [40]. Fe_2_O_3_ nanocubes were coated with a PDA layer to form Fe_2_O_3_@PDA core–shell nanocubes, followed by annealing at 500 °C to transform into Fe_2_O_3_@C core–shell structures. Then, the Fe_2_O_3_ core was partially etched to create a cavity inside by using hydrochloric acid. Yi Cui and co-workers have demonstrated the synthesis of sulphur@TiO_2_ yolk–shell nanostructures through a partial dissolution of sulfur core in toluene [41]. The sulphur particles were coated with TiO_2_ through hydrolysis of titanium diisopropoxide bis(acetylacetonate) in solution to form sulphur@TiO_2_ core–shell structures. The following partial dissolution of sulfur in toluene creates an empty space between the sulfur core and the TiO_2_ shell, resulting in the yolk–shell shape. The internal void space can accommodate the volume expansion of sulphur by preserving the sulfur core from cracking and fracture inside the shell. 

**Figure 3:**
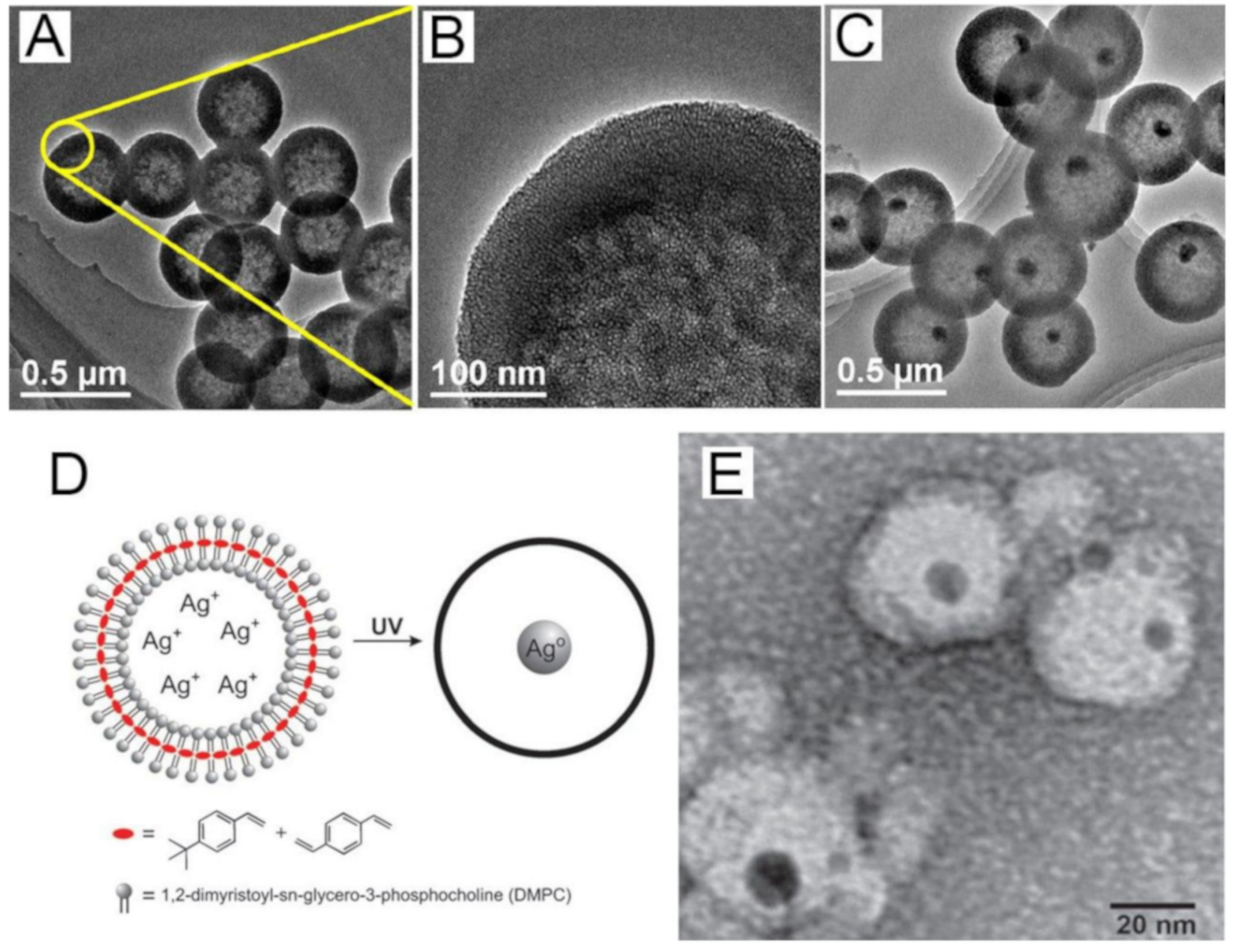
Self-templating syntheses for yolk–shell nanostructures. (**A**–**C**) TEM images of the hollow mesoporous silica (HMS) containing Fe nanoparticle prepared by a sequential two solvents impregnation-reduction reaction. Reproduced with permission from [44]. Copyright American Chemical Society, 2014. (**D**) Preparation of polymer nanocapsules loaded with Ag nanoparticles through a free-radical photoinitiator. (**E**) TEM image of nanocapsules containing the Ag yolk particles. Reproduced with permission from [45]. Copyright The Royal Society of Chemistry, 2010.

Yolk-shell morphology can also be synthesized through injecting the core material in a hollow shell from outside of the shell via transport along the pores. In this approach, the hollow shell act as the nanoreactors to confine and control the synthesis of the core, leading to the core–shell morphology with cavity [42]. Chen and co-workers have demonstrated the upconverting luminescent yolk–shell nanostructures by incorporating lanthanide-doped upconversion nanoparticles into hollow mesoporous silica [43]. Firstly, hollow mesoporous silica shell was prepared by a selective etching approach with an aid of a cationic surfactant. Then, the lanthanide-doped upconversion nanoparticles were synthesized within the hollow silica by loading precursor and calcination process to generate the core material inside. Lianjun Wang and co-workers have also demonstrated the yolk–shell nanostructure through an impregnation into a pre-formed hollow mesoporous silica nanosphere (Figure 3A,B) [44]. Fe@SiO_2_ yolk–shell nanospheres were synthesized by a sequential two-solvents impregnation-reduction method. Figure 3C shows the TEM image of the Fe@SiO_2_ yolk–shell nanosphere after the impregnation-reduction reaction. Lipid vesicles are also great nanoreactors for the synthesis of yolk–shell nanostructures through loading the core precursor in a hollow shell. Pinkhassik and co-workers reported the yolk–shell nanostructured Ag@polymer by using liposomes as the nanoreactors (Figure 3D,E) [45]. Liposomes with monomers and a photoinitiator in the bilayer and silver ions in the aqueous core were prepared by hydrating a mixture of lipids and monomers with a silver nitrate solution. The polymerization begins at the application of UV light and Ag nanoparticles starts to form with the aid of a photoinitiator. Finally, Ag@polymer yolk–shell nanostructure can be obtained. Likewise, Au@polymer yolk–shell nanostructures were also reported by loading reductant in a hollow nanocapsules [46]. Core contraction has also been studied as another promising strategy for synthesizing yolk–shell nanostructures. For example, the SnO_2_ yolk–shell particles were synthesized by spray pyrolysis at high temperature. The carbonization of sucrose within the precursor droplets changed into C–SnO_2_, followed by coating of SnO_2_ layer to form C–SnO_2_@SnO_2_ core–shell structures. Then, the combustion process induced the contraction of the C–SnO_2_ core to create void in the SnO_2_ shell. Other than the above methods, there have been other approaches to synthesize yolk–shell nanostructures such as surface-protected etching, fluid leakage, and hot-water-induced dissolution and reassembly [47,48,49].

## 3. Yolk–Shell Nanostructured Anodes 

For the next-generation Li rechargeable batteries, the most ideal anode material for lithium rechargeable batteries is lithium meal in terms of the lowest anode potential (−3.04 V vs. SHE), high specific capacity (3860 mAh/g), lightweight (0.53 g/cm^3^), and no demand for Cu current collector [50]. The uncontrolled dendrite formation in Li metal anodes has become a hurdle against the practical employment [51]. The sharp Li dendrite can grow through the separator during the cycle, thus leading to an internal short circuit. Recent research to tackle this problem relies on solid electrolyte interphase stabilization/modification by adding additives or conducting scaffold to form stable SEI [52,53]. The safety issue is still not fully resolved for mass production in industries. As an alternative, lithium alloy-based materials have been investigated due to their higher theoretical capacities (4200 mAh/g for Si, 1,623 mAh/g for Ge, 994 mAh/g for Sn, and 2,235 mAh/g for Al) and low operating potentials (~0.5 V vs. Li/Li^+^ for Si) than graphite leading to greater energy and power densities [54]. However, alloy-based anodes experience catastrophic capacity fading because of the large volume change upon electrochemical cycling (e.g., Δ*V*_Si_ = ~400% for full lithiation), which, in turn, may cause electrode pulverization and loss of contact with the current collector.

Despite the second highest abundance of silicon in nature and, thus, viable mass production, graphite anodes still dominate the marketplace due to major challenges preventing its widespread use. First of all, alloy anodes, including Si, undergo significant volume change, which leads to pulverization of the initial particle morphology and loss of electrical contact. Secondly, the low electrochemical potential of alloy-type anodes provokes thick SEI film due to the reductive decomposition of the organic electrolyte [55]. In addition, the SEI rupture from volume change and particle fracture during cycling and an electrolyte is continually consumed by exposing the electrode surface to the electrolyte. This excessive growth of SEI lows Coulombic efficiency by enhancing resistance to ionic and electronic transport. The stabilization of the SEI layer was obtained by surface coating with metal oxide and carbon [56,57,58]. However, those surface coatings cannot withstand the large volume changes of Si during discharge/charge cycles. Keeping the advantage of the surface coating, a new approach has recently been tried to give spatial room for active material to maintain its initial morphology despite the volume change by creating yolk–shell nanostructures [17,18,19]. Conversion-based anode materials also experience the large volume change during Li or Na insertion/extraction process leading to fast capacity fading. Transition metal oxides and sulfides have been prepared as yolk–shell nanostructures to provide stable SEI formation and increased electronic conductivity, especially when they are covered with carbonaceous materials. In this section, we will discuss the recent development of metals, metal oxides, and metal sulfides based on yolk–shell nanostructures for Li or Na rechargeable batteries. Table 1 shows various lithium/sodium rechargeable anode materials based on yolk–shell nanostructures.

### 3.1. Alloy-Type Materials

Alloying/de-alloying anode materials in LiBs and NIBs are attractive for their very high capacities. However, the large volume change upon electrochemical cycling is detrimental to commercializing them, so that several strategies have been developed: designing nanostructures and fabrication of composites with lithium material [110]. To alleviate the fast capacity fading due to electrode pulverization, capping materials, such as conductive carbon or porous metal oxides, can be exploited as a buffer layer to endure the large volume exchange during cycling. Core–shell and yolk–shell nanostructures are the examples in a way to provide the robust wall and surround the active electrode part. In terms of guaranteeing spacious room for nanostructured anode materials to keep their morphologies, yolk–shell nanostructures are advantageous compared to core–shell, which is necessary for alloy-type anode materials due to their very large volume change.

#### 3.1.1. Silicon

Nanostructured Si anodes such as nanoparticles, nanowires, and nanotubes can relieve the huge morphological change and shorten the diffusion path of lithium ions. Nevertheless, pure Si nanomaterials tend to aggregate during lithiation/delithiation, leading to aggravation of electronic transport [111]. Hybridization of the nanostructured Si with electrochemically inactive matrix is an alternative to pure Si nanostructures. The inactive materials can stabilize Si nanomaterials and prevent the aggregation by alleviating the mechanical stress from huge volume change [112]. Unlike core–shell structured anodes, novel yolk–shell nanostructures have, recently, been on the focus of battery anode materials because the active core (yolk) can expand upon lithiation without breaking the shell and stabilize the SEI layer [18]. Especially, encapsulating Si nanoparticles with carbon has been the main idea of fabricating yolk–shell nanostructured Si anode due to its elastic nature and electronic conductivity. Many preparation methods include chemical etching (acid or base) the unnecessary silica or calcium carbonate layer after carbonization to create a void in the yolk–shell nanostructure [17,18,19,60,61,62,63,65].

**Figure 4:**
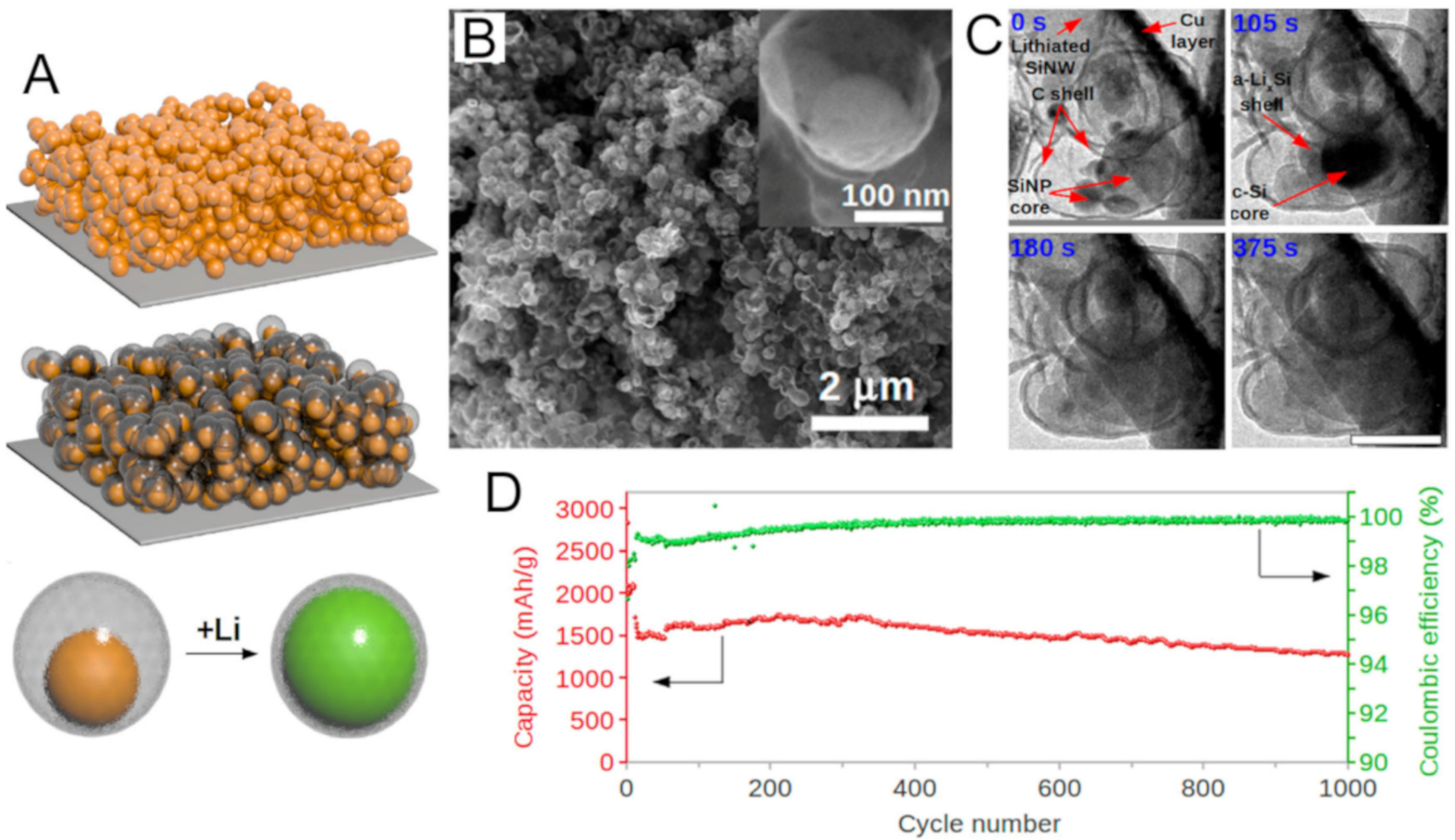
Si@C yolk–shell nanostructure. (**A**) Comparison of a conventional slurry coated silicon nanoparticle and Si@C yolk–shell electrodes. SEI on the surface of the Si nanoparticles is not stable, leading to failure of the battery in a conventional slurry electrode. The yolk–shell nanostructure creates enough space and allows the Si to expand without rupturing the shell layer. (**B**) SEM image of Si@C yolk–shell nanostructure. (**C**) Serial in situ TEM images showing the expansion of the Si yolk part. The scale bar is 200 nm. (**D**) Delithiation capacity and Coulomb efficiency of the first 1000 galvanostatic cycles between 0.01−1 V at 1 C rate. Reproduced with permission from [18]. Copyright American Chemical Society, 2012.

Cui and co-workers have, successfully, designed a Si@C yolk–shell nanostructure and demonstrated prolonged cycle life by characterizing the in-situ Si nanoparticle expansion during electrochemical lithiation [18]. A successful design for Si anode requires the following prerequisite: nanostructuring of silicon, stable SEI, well-controlled pore, and up-scaled fabrication. Figure 4A shows a comparative scheme between a conventional slurry Si nanoparticle electrode and Si@C yolk–shell electrode. The expansion of Si nanoparticles disrupts the microstructure of the electrode during lithiation in the case of the conventional slurry electrode. The void in the yolk–shell nanostructure allows Si to expand without rupturing the carbon coating layer, which enables a stable and thin SEI layer formation on the outer surface of the carbon. Furthermore, the volume change of the Si does not break the outer shell. The Si@C yolk–shell nanostructure was fabricated by coating, conformally, Si with SiO_2_ sacrificial layer and then polydopamine, followed by carbonization with nitrogen doping and selective removal of SiO_2_ layer in acidic solution (Figure 4B). This completely sealed structure was monitored with in-situ TEM to demonstrate that the Si@C yolk–shell provides excellent electrochemical cycling performance to alleviate the severe volume change of Si during lithiation/delithiation (Figure 4C). Pristine Si nanoparticles (0 s) are visible within the outer C shell. The volume expansion of Si nanoparticles is seen in 105 s to produce the partially lithiated Li_x_Si shell/crystalline Si core in the carbon shell. Full lithiation increases the size of Si particles up to ~300 nm. Furthermore, the thickness of carbon shell increases from 5 to ~20 nm after lithiation implying that the carbon coating is also lithiated and creates a thin layer of ionic liquid electrolyte at the surface. Figure 4D shows the reversible capacity of Si@C yolk–shell electrode reached 2833 mAh/g for the initial cycle at C/10 and stabilized at ~1500 mAh/g at 1 C. No capacity retention was observed in the first 300 cycles and 74% of the capacity was achieved after 1000 cycles. In contrast, the bare Si nanoparticle and Si@C core–shell electrodes showed very fast capacity fading in the first 10 cycles. The stable SEI formation of the Si@C yolk–shell electrode was evident in the Coulombic efficiency profile. 

**Figure 5:**
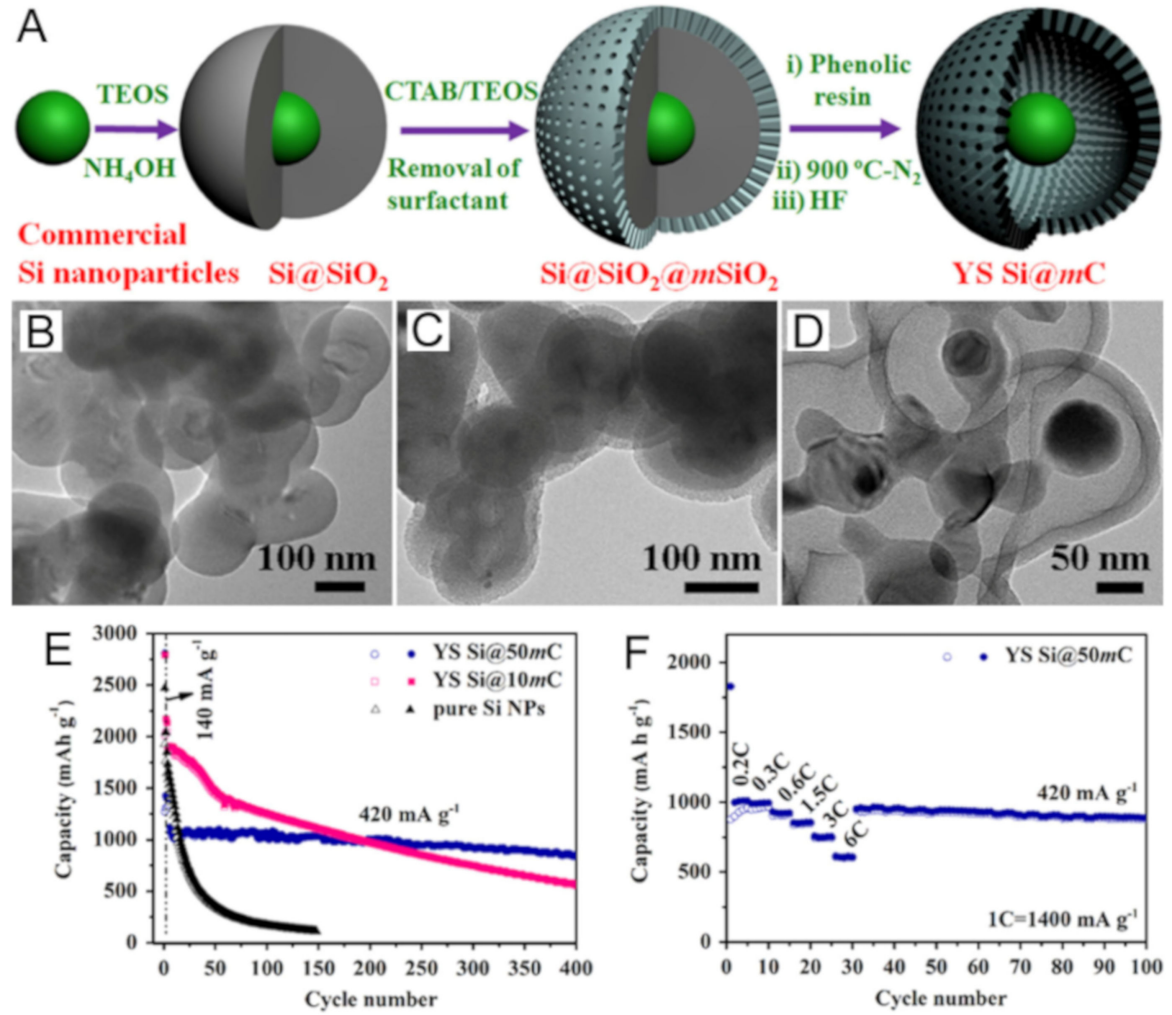
(**A**) Schematic process for the formation of the mesoporous carbon-coated silicon yolk–shell nanostructure. TEM images of (**B**) Si@SiO_2_ core–shell nanoparticles, (**C**) Si@SiO_2_@mSiO_2_ core–shell nanoparticles, and (**D**) Si@mC yolk–shell nanostructure. (mSiO_2_: mesoporous SiO_2_, mC: mesoporous carbon) (**E**) Charge–discharge cycling performance of Si nanoparticle and carbon-coated yolk–shell nanostructure electrodes. (**F**) Rate performance of yolk–shell nanostructure electrode. Reproduced with permission from [63]. Copyright Elsevier Ltd, 2015.

Open-ended mesoporous carbon shell was devised to facilitate the fast diffusion of Li^+^ ions and provide the full immersion of Si core materials in the electrolyte for higher rate capability [63]. Si@mC (mesoporous carbon) yolk–shell nanostructures are developed by Zhang and co-workers by using mesoporous SiO_2_ as a template, which is etched away later, to form mesoporous carbon shell containing Si nanoparticle in the core (Figure 5). For a comparative study, they prepared two types of Si@mC according to the void space inside the carbon shell (10 and 50 nm). The electrochemical cycling properties of Si@mC yolk–shell electrodes outperform the pure Si nanoparticle electrode (Figure 5E). Another notable observation is that enough space inside the carbon shell is important in terms of cycling performance and rate capacity retention. Small void is hard to afford the large volume expansion of Si, which can result in structural collapse of the carbon shell. Thus, the successful design of yolk–shell nanostructure takes into consideration the efficient volume expansion of Si core material. Other than the chemical etching method, spray pyrolysis is another approach to form yolk–shell Si nanostructures, i.e., Si@C, Si@NiO [59,64]. 

#### 3.1.2. Tin and Tin Oxide

**Figure 6:**
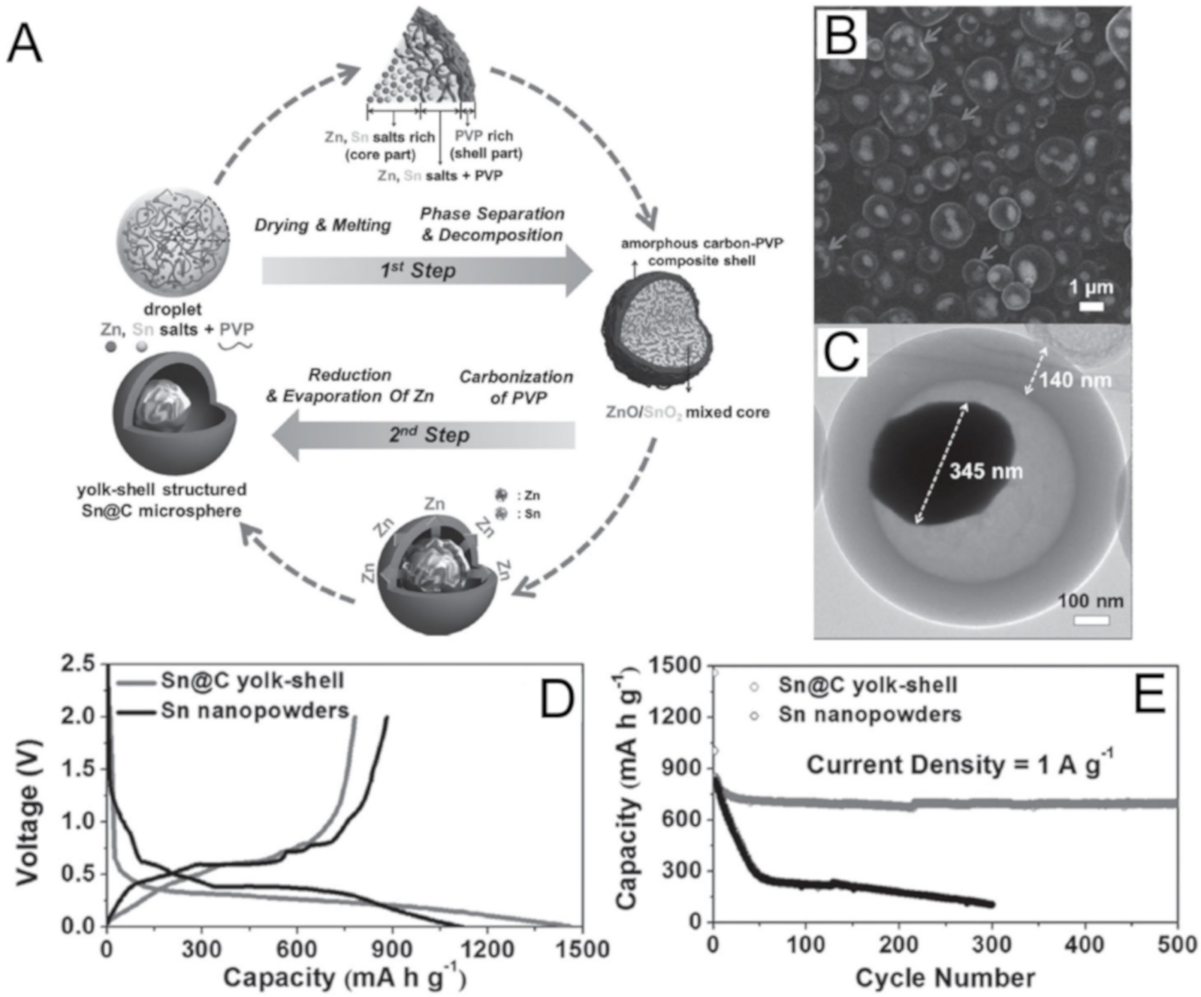
(**A**) Schematic illustration of the formation mechanism of yolk–shell Sn@C nanostructure (in the middle). SEM (**B**) and TEM (**C**) image of the yolk–shell Sn@C spheres by eliminating Zn component. (**D**) Initial charge and discharge profiles of tin powder and Sn@C yolk–shell electrodes. (**E**) Cycling performance of the two electrodes. Reproduced with permission from [66]. Copyright Wiley-VCH, 2015.

As an alloy-type anode alternative, tin is also a promising material due to its high specific capacity and low operating potential. Although tin cannot beat silicon in terms of gravimetric capacity (4200 vs. 991 mAh/g, based on the formation of Li_4.4_Sn, Sn + 4.4Li^+^ +4.4e^−^ ↔ Li_4.4_Sn), it has a comparable volumetric capacity of 2020 mAh/cm^3^ (2400 mAh/cm^3^ for Si) and better electrical resistivity than graphite [113]. Nevertheless, tin is prone to capacity deterioration during charging/discharging, like other alloy-type anode materials, due to huge volume change (~300%) leading to electrode pulverization, delamination from the current collector, and SEI formation. Designing pure tin anode as nanostructure has been suggested to alleviate the stress of tin from volume expansion [113]. Intrinsic volume change during lithiation/delithiation pushes the research towards using the buffer layer to keep tin nano-/micro-scale anode from pulverization by creating core-protected structure. Kang and co-workers have demonstrated that yolk–shell nanostructured Sn@C electrode showed better cycling stability than pure Sn powder [66]. Carbon-coated Sn microsphere was prepared by spray pyrolysis (Figure 6). Firstly, SnO_2_–ZnO@C microsphere (core–shell) was synthesized and, then, transformed to yolk–shell Sn@C microsphere by reducing SnO_2_ and vaporizing Zn (low vaporizing temp. of Zn, ~900 °C). H_2_/Ar mixture gas reduced SnO_2_ and ZnO and the undecomposed PVP created a carbonized shell at 1000 °C. Figure 6C shows the reduced Sn yolk covered with carbon shell. The yolk–shell Sn@C electrode showed initial discharge and charge capacities of 1458 and 781 mAh/g indicating an initial Coulombic efficiency of 54%. In comparison, the pure tin nanopowder electrode has 75% of initial Coulombic efficiency due to a large irreversible capacity from an amorphous carbon shell in yolk–shell electrode. However, the yolk–shell Sn@C electrode showed a better long-term cycling performance in Figure 6E. The capacity retention of Sn@C electrode is 83% from the second cycle to 500^th^ cycle. The stable cycling is attributed to the void in yolk–shell nanostructure, in which Sn can maintain its morphology inside the carbon shell during lithiation/delithiation. The concept of Sn@C yolk–shell nanostructure was also demonstrated in the form of nanotube [67], integration with nanofiber [68,71], and core-sheath nanowire [69]. 

Tin oxide has the lowest operating voltages (0.3 and 0.5 V vs. Li/Li^+^, for discharge and charge) among other transition metal oxides. Tin oxide is also classified as alloy-type anode materials according to the following electrochemical processes: [114]

SnO_2_ + 4Li^+^ + 4e^−^ ↔ Sn + 2Li_2_O (1)

Sn + *x*Li^+^ + *x*e^−^ ↔ Li*_x_*Sn (0 ≤ *x* ≤ 4.4) (2)

**Figure 7:**
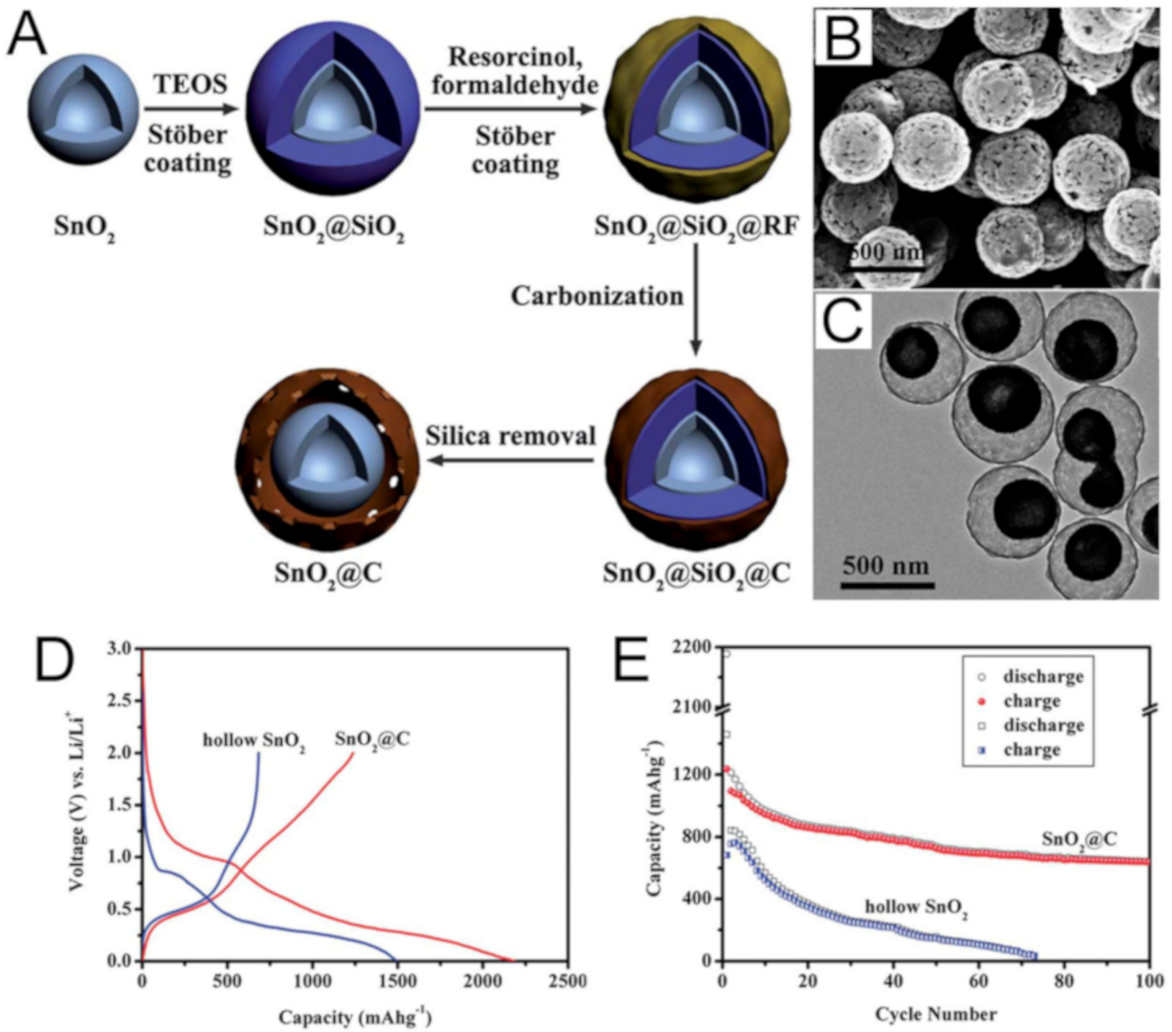
(**A**) The formation process of the SnO_2_@C yolk–shell nanostructure. SEM (**B**) and TEM (**C**) images of the SnO_2_@C yolk–shell nanostructures. Initial charge–discharge curves (**D**) and cycling performance (**E**) of the SnO_2_@C yolk–shell nanostructure and hollow SnO_2_ particle electrodes. Reproduced with permission from [72]. Copyright The Royal Society of Chemistry, 2014.

Thus, tin oxide is one of the alloy-type anode materials that has been investigated to design yolk–shell nanostructure for stable long-term cycling. Mostly, tin oxide nanoparticles were coated with a carbonaceous shell, followed by etching the template or buffer layer and carbonization at high temperatures. Well-controlled SnO_2_@C yolk–shell nanospheres were reported by Zhao and co-workers (Figure 7) [72]. Uniform SnO_2_@C nanospheres were synthesized by coating silica layer and resorcinol-formaldehyde (RF). The silica layer was etched and RF was carbonized to form hollow SnO_2_@C yolk–shell nanostructure (Figure 7A). Figure 7B,C shows the SEM and TEM images of hollow SnO_2_@C nanospheres after carbonization. The size of the void space and the thickness of the carbon shell can be controlled by controlling the precursors of silica and carbon. Due to the carbon shell, the first discharge/charge capacities of SnO_2_@C exhibit a larger value of 2190 and 1236 mAh/g than those of pure SnO_2_ electrode, respectively (Figure 7D). Despite the lower first Coulombic efficiency of SnO_2_@C (43%), the yolk–shell nanostructure shows the improved cycling performance with a high reversible capacity of ~950 mAh/g after 10 cycles and ~630 mAh/g after 100 cycles (Figure 7E). In contrast, the pure SnO_2_ electrode approaches to zero capacity after 70 cycles indicating that the carbon shell and void in yolk–shell SnO_2_@C facilitate the cycling stability.

#### 3.1.3. Aluminum

**Figure 8:**
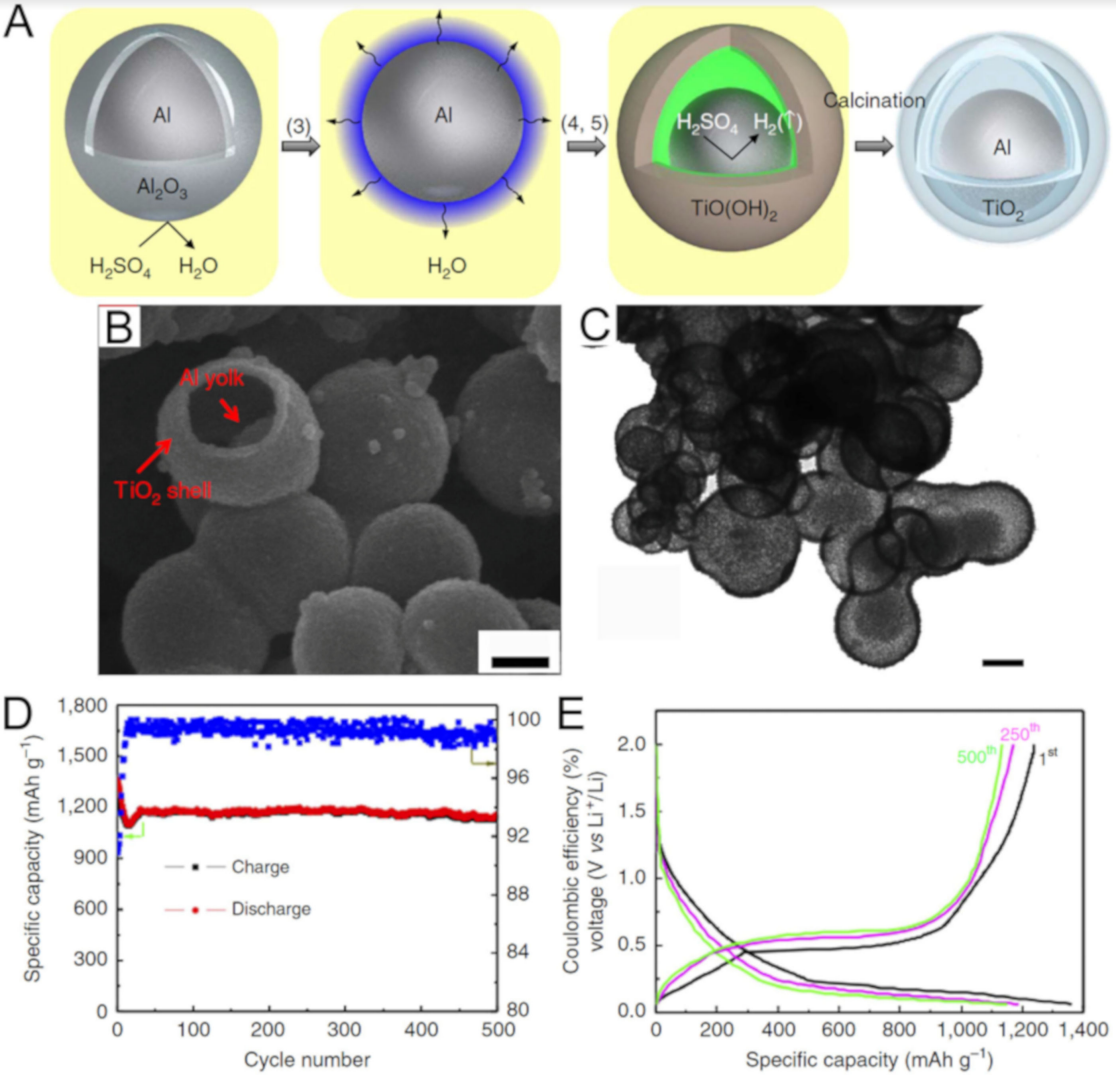
(**A**) Synthetic step of Al@TiO_2_ yolk–shell nanospheres. SEM (**B**) and TEM (**C**) images of yolk–shell Al@TiO_2_ nanostructures. (**D**) Cycling performance and Coulombic efficiency at 1 C rate. Scale bars are 20 nm (**B**) and 10 nm (**C**). (**E**) Charge–discharge profile of the first, 250^th^, and 500^th^ cycling. Reproduced with permission from [77]. Copyright Springer Nature Publishers Ltd, 2015.

Aluminum is another attractive alloy-type anode material owing to cheap price ($2000/ton), high theoretical capacity (2235 mAh/g, based on 4Al + 9Li^+^ + 9e^−^ ↔ Li_9_Al_4_), and high electrical conductivity [115]. However, the practical performance is still suffering from relatively low capacity due to the electrode damage from the volume change (~100%) and pulverization. Even the hybrid structure of aluminum nanoparticles with carbon achieved only ~900 mAh/g [116]. Instead of carbon shell, inorganic and mesoporous TiO_2_ shell was employed as a constituent of yolk–shell Al@TiO_2_ anode material by Li and co-workers [77]. The yolk–shell Al@TiO_2_ nanospheres were synthesized through forming TiO(OH) and etching Al_2_O_3_ on the surface of aluminum particles to create a void inside the shell. TiO(OH) shell is converted to anatase TiO_2_ by calcination at 450 °C (Figure 8A). The resultant yolk–shell Al@TiO_2_ nanospheres have an aluminum core (~30 nm) and TiO_2_ shell (~3 nm) with a controllable void space (Figure 8B,C). The Al@TiO_2_ yolk–shell nanosphere electrode has a long lifecycle, which maintains ~1100 mAh/g after 500 cycles at 1 C rate with 93% of Coulombic efficiency (Figure 8D,E). Even at 10 C rate, the electrode achieved a capacity of 661 mAh/g after 500 cycles. The high rate performance is attributed to the good electrical conductivity of aluminum. Compared to silicon-carbon yolk–shell electrodes, Al@TiO_2_ has lower capacity at a low rate (1 C), but exhibits higher rate performance after a long cycle. The Al@TiO_2_ has a few times larger specific capacity than LTO and graphite.

### 3.2. Conversion Materials

Conversion-based anode materials follow the electrochemical reaction mechanism to form transition metal compounds such as oxides, phosphides, sulfides, selenides and nitrides with lithium or sodium. These materials involve the reduction (oxidation) of the transition metal along with the composition (decomposition) of lithium or sodium compounds (Li*_x_*X*_y_*, Na*_x_*X*_y_*; X = O, P, S, Se, N). A high number of electrons in the conversion reaction of these materials can induce high reversible capacities. Nevertheless, metal oxides have some issues such as very low electrical conductivity, unstable SEI formation, and poor capacity retention due to phase segregation, volume change, and ionic/electronic transport [9]. Thus, as a buffer layer or conducting layer, yolk–shell nanostructure has been employed to improve electrochemical properties of conversion-type anode materials. Synthetic approaches can be classified into four mechanisms based on the step of void formation: spray pyrolysis, Ostwald ripening, etching, and thermal treatment.

**Figure 9:**
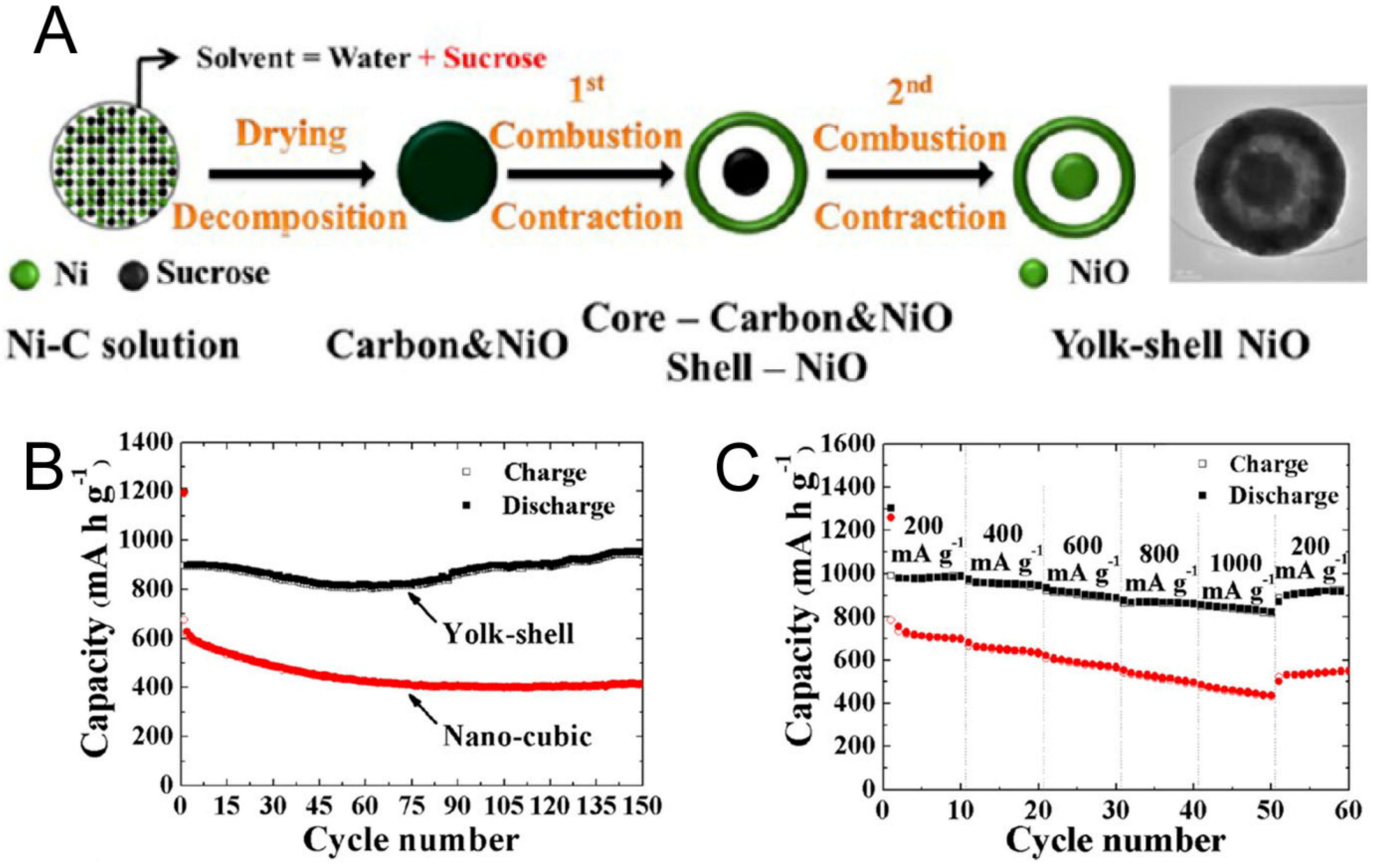
(**A**) Schematic diagram of the yolk–shell NiO nanosphere by spray pyrolysis. Cycling performance (**B**) and rate performance (**C**) of the yolk–shell and single crystalline cubic NiO nanopowder electrodes in the voltage range of 0.001−3 V. Reproduced with permission from [91]. Copyright American Chemical Society, 2014.

Spray pyrolysis is a continuous and rapid one-pot process for fabricating micro-/nanopowder. In addition, neither template material nor etching step is necessary compared to the templating method. Various transition metal oxides can be synthesized by spray pyrolysis with high crystallinity for a few seconds. The morphologies of the yolk–shell particles prepared by spray pyrolysis were affected by the types of spray solution and temperature. The formation of carbon-metal oxide particles was formed as an intermediate product, followed by decomposition of precursors and combustion of carbon to create yolk–shell nanostructure. Thus, most yolk–shell particles synthesized by spray pyrolysis have the same composition of shells with core materials. Figure 9 shows the formation of yolk–shell NiO@NiO nanoparticle, demonstrated by Kang and co-workers [91]. A dense carbon/NiO intermediate particle was formed from Ni-Sucrose droplet by the decomposition of nickel nitrate and the carbonization of sucrose. Further combustion of the intermediate particle produced core–shell carbon/NiO@NiO composite and, finally, yolk–shell NiO@NiO particles after carbon combustion. For a comparative study, the electrochemical measurements were performed with both the yolk–shell NiO@NiO and single-crystalline cubic NiO particles. Figure 9B shows the cycling performances of the two electrodes at 1 C. Whereas the cubic NiO electrode showed no increase in discharge capacities after 150 cycles, the yolk–shell electrode experienced capacity increase after 60 cycles. This is attributed to the formation of a gel-like film of the transition metal oxides due to small grain and size [117]. The rate performance of the yolk–shell NiO@NiO is slightly better than the pure cubic particle (Figure 9C). The simple and self-templating spray pyrolysis have been applied to fabricate various metal oxides yolk–shell nanostructure; Co_3_O_4_ [78], CoMn_2_O_4_ [79], Fe_2_O_3_ [83], NiMoO_4_ [92], ZnCo_2_O_4_ [97], ZnFe_2_O_4_ [98], ZnO/Mn_3_O_4_ [99], MoSe_2_ [102] and mixed metal oxides [118]. Furthermore, chemical transformation can produce transition metal sulfide yolk–shell nanostructure by an anion exchange. The prepared yolk–shell SnO_2_ particles can be converted into yolk–shell SnS particles through sulfidation [103].

**Figure 10:**
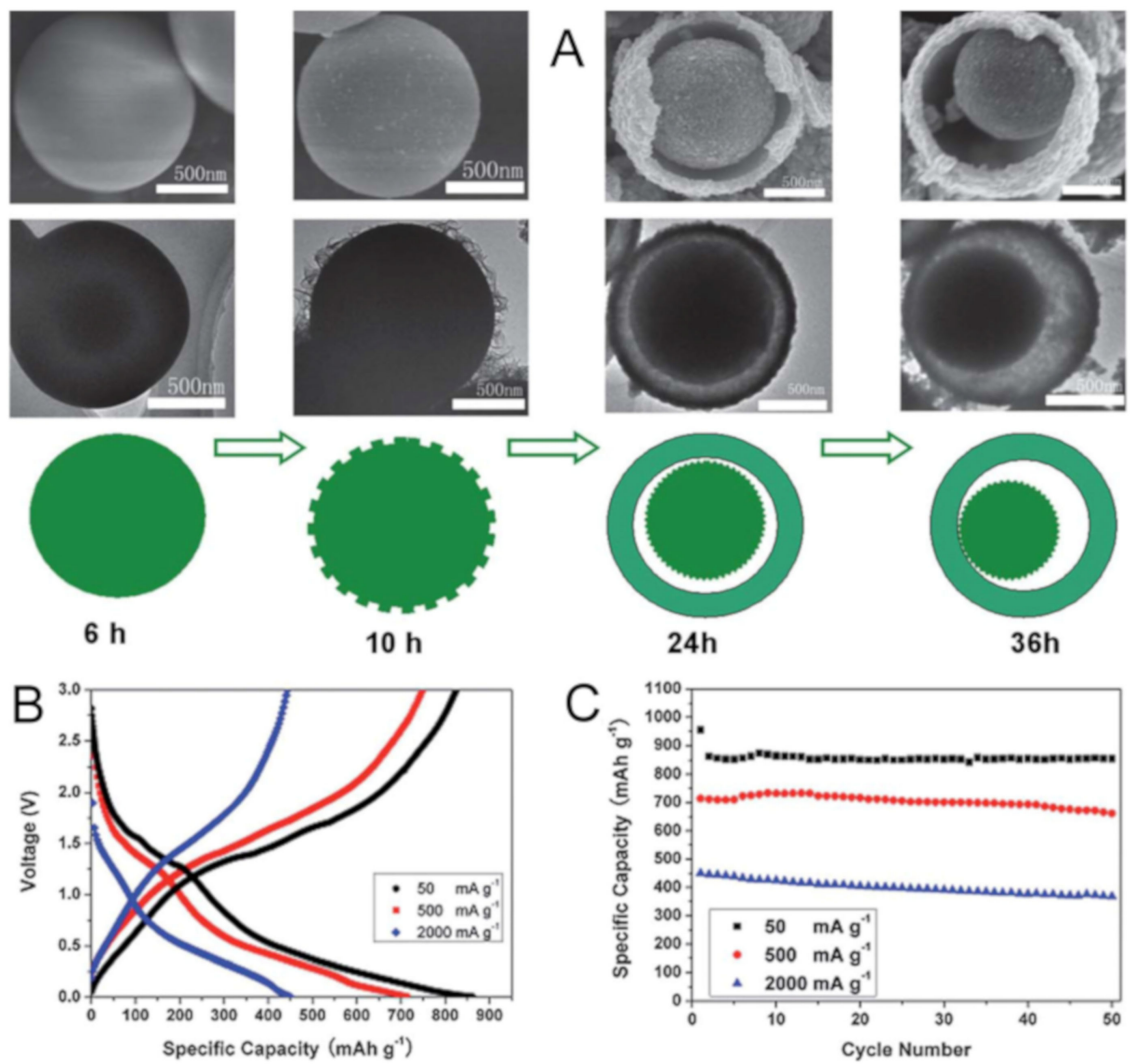
(**A**) Schematic illustration of the formation process of yolk–shell MoO_2_ spheres by Ostwald ripening and the corresponding SEM and TEM images. (**B**) Charge–discharge voltage profiles of the yolk–shelled MoO_2_ electrode at different current densities. (**C**) Cycling performance. Reproduced with permission from [87]. Copyright The Royal Society of Chemistry, 2013.

Ostwald ripening is another approach to form yolk-shell nanostructure without template material. Generally, a solid material starts to dissolve or evacuate and a shell is formed on the surface of the solid core at the expense of dissolving the core material due to the high surface energy. Figure 10A shows the sequential steps of the formation of the yolk–shell MoO_2_ microsphere through Ostwald ripening. From the SEM images, the smooth microsphere starts to form small nanoparticles on the surface and the growth of the shell, followed by creating void inside. The resultant yolk–shell MoO_2_ is crystalline with monoclinic MoO_2_ phase. The rate performance and cycling stability of the yolk–shell MoO_2_ electrode is reasonable, which has 714 and 450 mAh/g at 0.5 A/g and 2 A/g, respectively (Figure 10B,C). This enhanced electrochemical performance of the MoO_2_ microspheres can be originated from the better kinetics of the yolk–shell nature, which facilitates the electrolyte to transport Li ions during intercalation/deintercalation. Other anode materials structured as yolk–shell are also investigated by Ostwald ripening; TiO_2_ [106], V_2_O_3_ [94], ZnO [95], ZnO@ZnO/NiO [96], CoS_2_ [100].

**Figure 11:**
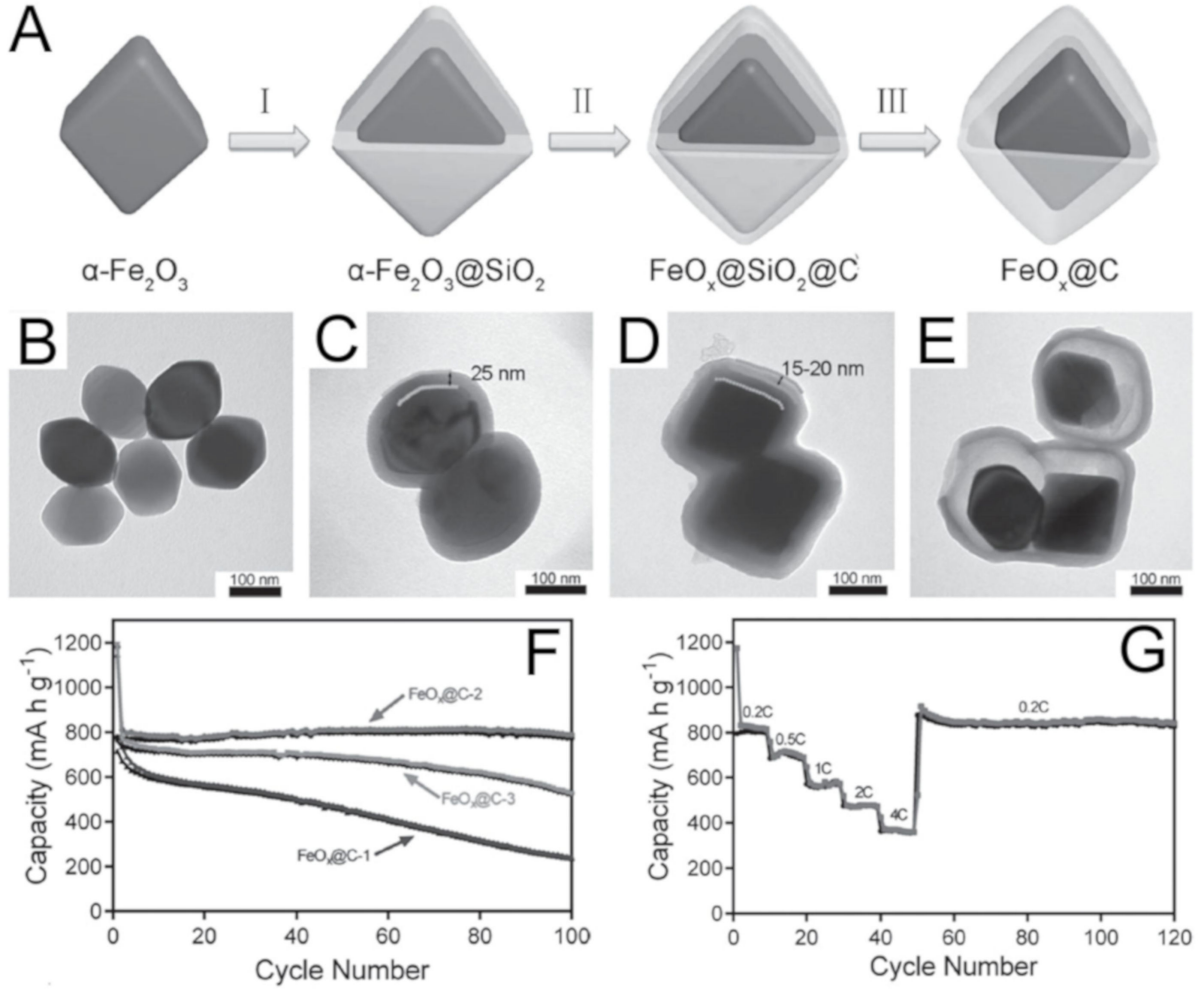
(**A**) Schematic illustration showing the formation of FeO*_x_*@C yolk–shell nanostructure. (**B**–**E**) TEM images corresponding to each step in (**A**): *α*-Fe_2_O_3_ → *α*-Fe_2_O_3_@SiO_2_ → FeO*_x_*@SiO_2_@C → FeO*_x_*@C. (**F**) Cycling capacity of FeO*_x_*@C yolk–shell nanostructures at 0.2 C. (**G**) Rate performance of FeO*_x_*@C-2. Reproduced with permission from [82]. Copyright Wiley-VCH, 2014.

Templating approach towards yolk–shell nanostructure design is beneficial in a way that heterogeneous shell materials can be coated around the core, followed by the elimination of unwanted materials. One of the most historic methods to use template material for yolk-shell nanostructure is coating the first layer and then etching that layer after coating the second layer. Most Si@C yolk–shell nanostructures were achieved by the etching method. Likewise, metal oxides can also be produced as a yolk–shell particle through the etching method. An iron oxid@carbon yolk–shell nanostructure was designed through etching by Yu and co-workers [82]. *α*-Fe_2_O_3_ nanocrystals were coated with a conformal silica layer by controlled hydrolysis and condensation of TEOS to form *α*-Fe_2_O_3_@SiO_2_ (Figure 11A). Poly-dopamine was coated on the surface, followed by carbonization to obtain FeO*_x_*@SiO_2_@C nanoparticles. Basic solution was used to remove the sacrificial silica layer. The resultant FeO*_x_*@C yolk–shell nanoparticles have a controllable void size depending on the thickness of the silica layer (Figure 11B–E). The optimal void size of FeO*_x_*@C yolk–shell anode material was investigated by performing electrochemical tests. Figure 11F shows the cycling performance of the yolk–shell electrode indicating that FeO*_x_*@C-2 (silica layer thickness: 15~20 nm) achieved the highest and stable long-term capacity value, compared to the thinner (~9 nm) and thicker layer (~45 nm). FeO*_x_*@C-2 electrode delivers a high capacity of 820 mAh/g at 0.2 C and ~380 mAh/g at 4 C, which is comparable to the theoretical capacity of graphite (Figure 11G). FeO*_x_*@C-2 outperforms the other FeO*_x_*@C electrodes since the small void space was found to rupture the carbon shell after cycling, which is indicative of insufficient space for volume expansion. However, in the case of the thicker FeO*_x_*@C nanoparticles, volumetric capacity becomes lower. Templating methods through etching has produced other metal or metal oxide yolk–shell nanostructures; Fe_2_O_3_@C [80], Fe_3_O_4_@C [81], MnO_2_@C [86], Ni@graphene [89].

**Figure 12:**
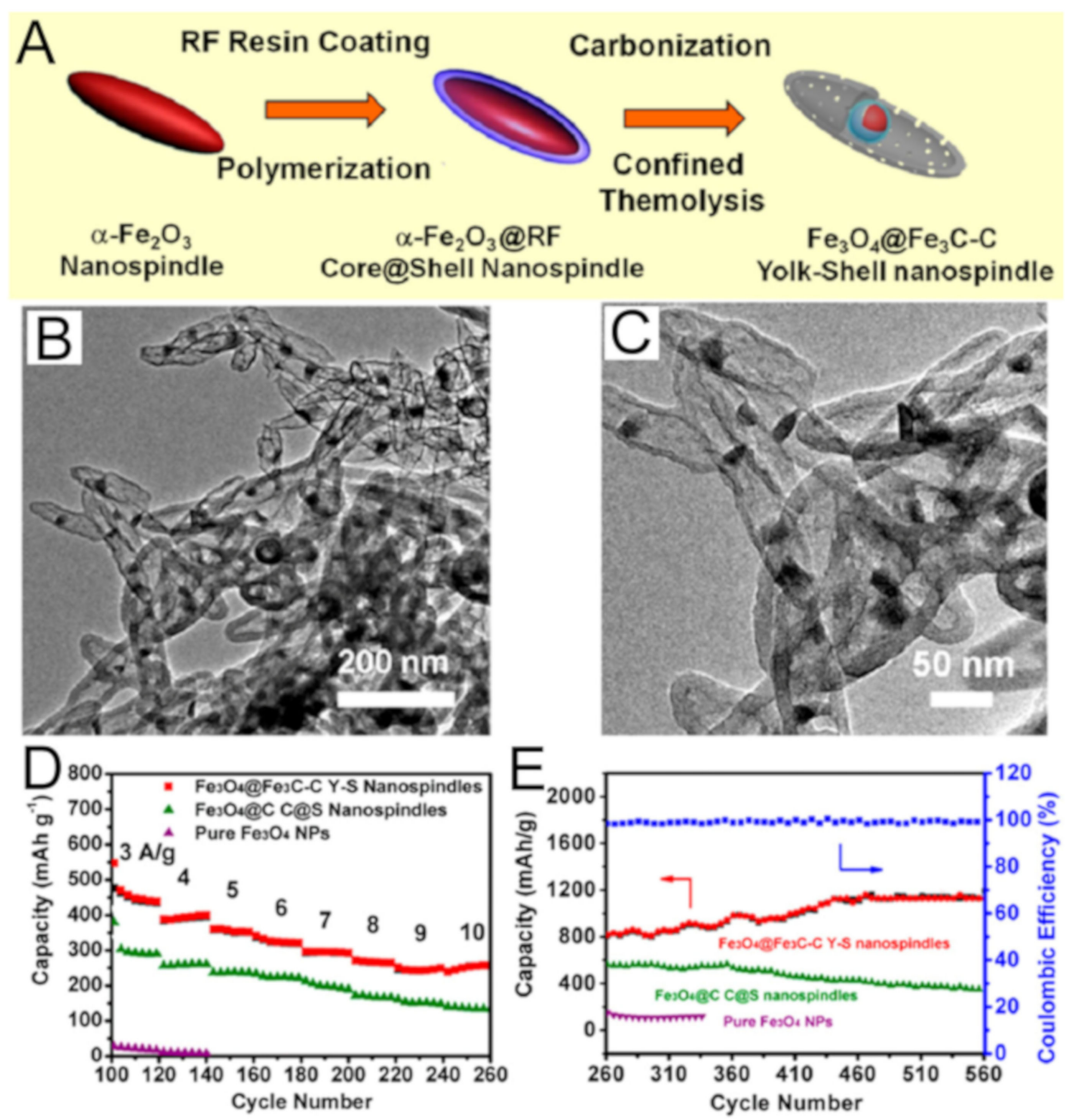
(**A**) Scheme showing the formation of Fe_3_O_4_@Fe_3_C-C yolk–shell nanostructure. (**B,C**) TEM images of Fe_3_O_4_@Fe_3_C–C. (**D**) Capacity retention of the yolk–shell electrode, Fe_3_O_4_@C core–shell electrode, and pure Fe_3_O_4_ nanoparticle electrode. (**E**) Cycling performance of the three electrodes and Coulombic efficiency of the yolk–shell electrode. Reproduced with permission from [47]. Copyright American Chemical Society, 2015.

Yolk–shell nanostructures containing carbon shell can be obtained by thermal treatment to carbonize a carbonaceous shell material, followed by contraction of core materials to create a void. Fe_3_O_4_/Fe_3_C@C yolk–shell nanospindles were synthesized by Guo and co-workers. Firstly, uniform *α*-Fe_2_O_3_ nanospindle were prepared by hydrothermal reaction and coated with a conformal RF resin layer to form *α*-Fe_2_O_3_@RF core–shell structure (Figure 12A). Thermal treatment at 550 °C produced Fe_3_O_4_/Fe_3_C@C yolk–shell nanostructure by escaping the core from the carbon shell (Figure 12B,C). Interestingly, only Fe_3_O_4_@C core–shell nanospindles were obtained when RF was replaced by another carbonaceous material (glucose). The electrochemical performance of Fe_3_O_4_/Fe_3_C@C yolk–shell nanospindles were compared with pure Fe_3_O_4_ nanoparticle and Fe_3_O_4_@C core–shell nanospindle in Figure 12D,E. The Fe_3_O_4_/Fe_3_C@C yolk–shell nanospindles outperforms the other two electrodes in terms of rate capability and long-term cycling stability. The increasing capacity of Fe_3_O_4_/Fe_3_C@C yolk–shell nanospindles in Figure 12D can be found in metal oxide electrodes [119,120]. Metal oxide yolk–shell nanostructures synthesized by thermal treatment include Cr_2_O_3_@TiO_2_ [47], Fe_3_O_4_@TiO_2_ [84], FeO*_x_*@graphene [44], MnO_2_@C [85], NiO@C [88] and TiO_2_@C [108].

## 4. Conclusions and Outlook

In this review, the yolk–shell nanostructures are covered in terms of designing strategies and applications in lithium and sodium battery anode materials. Compared to the conventional core–shell structure, the hollow shell containing the movable core possesses unique morphological features bringing low density, large surface area, and great loading capacity. Typically, the building-up of yolk–shell nanostructures can be classified into templating and self-templating approaches according to the use of the sacrificial layer and the necessity of the removal process. Templating methods usually exploit rigid or soft materials as sacrificial materials such as SiO_2_, carbon, metal, metal oxides, oligomers, and vesicles. While the templating approaches are straightforward due to the role of an additional layer, the self-templating methods cover a wide range of synthetic strategies such as galvanic replacement, Kirkendall effect, Ostwald ripening, partial removal of core, core injection, core contraction, surface-protected etching, and so on. The yolk–shell nanostructured materials have the potential for battery anode due to their morphological features relieving volume expansion and facilitating rapid diffusion with electron transportation. These superior merits of yolk–shell nanostructured anodes were demonstrated from the many reports on the improvement of Li and Na storage performance with high specific capacity, rate capability, and stable long-term cyclability. Nevertheless, further study is needed to focus on developing advanced morphologies for more precise control over the shape and function. In addition, the environmentally-friendly etching or dissolution process should be explored because the usual dissolution solvents like hydrofluoric acid are very harmful. Since sodium ions are larger than lithium ions, it is also required to find suitable materials with appropriate morphologies for the reasonable volume expansion ratio and high specific capacity. Definitely, the yolk–shell nanostructures with tailorable inner space are promising for improving battery performance in the near future only if a commercially available process is set up.

## Figures and Tables

**Table 1:** Summary of various Li/Na rechargeable anode materials constructed as yolk–shell nanostructures. (Pyrolysis: spray pyrolysis_,_ Thermal: thermal treatment, Ripening: Ostwald ripening, LTO: Li_4_Ti_5_O_12_).

Yolk	Shell	Fabrication	Pros/Cons
**Alloy/de-alloy materials**			
Si	C [17,18,19,59,60,61,62,63], NiO [64], SiO_2_@C [65]	Etching [17,18,19,60,61,62,63,65], Pyrolysis [59,64]	high capacity and energy density, good safety/poor cycling
Sn	C [66,67,68,69,70,71]	Thermal [67,68,69,70,71], Pyrolysis [66]	
SnO, SnO_2_	C [72,73,74,75], SnO_2_ [76]	Etching [72,73], Thermal [74,75,76]	
Al	TiO_2_ [77]	Etching [77]	
**Conversion materials**			
Co_3_O_4_, CoMn_2_O_4_	Co_3_O_4_ [78], CoMn_2_O_4_ [79]	Pyrolysis [78,79]	high capacity, high energy, environmentally-compatibility/low coulombic efficiency, poor cycling, unstable SEI formation, large potential hysteresis
Cr_2_O_3_	TiO_2_ [47]	Thermal [47]	
Fe_2_O_3_, Fe_3_O_4_, FeO_x_	C [47,80,81,82], grapheme [44], Fe_2_O_3_ [83], TiO_2_ [84]	Etching [80,81,82], Pyrolysis [83], Thermal [44,47,84]	
MnO, MnO_2_	C [85,86]	Thermal [85], Etching [86]	
MoO_2_	MoO_2_ [87]	Ripening [87]	
Ni, NiO, NiMoO_4_, NiCO_2_O_4_	C [88], graphene [89], NiO [90,91], NiMoO4 [92], NiCO_2_O_4_ [93]	Etching [89], Pyrolysis [91,92], Thermal [88,90,93]	
V_2_O_3_	V_2_O_3_@C [94]	Ripening [94]	
ZnO, ZnCo_2_O_4_, ZnFe_2_O_4_, ZnO–Mn_3_O_4_	C [95], ZnO–NiO [96], ZnCo_2_O_4_ [97], ZnFe_2_O_4_ [98], ZnO/Mn_3_O_4_ [99]	Pyrolysis [97,98,99], Ripening [95,96]	
CoS_2_, Co_9_S_8_	CoS_2_ [100], Co_9_S_8_ [101]	Ripening [100], Pyrolysis [101]	
MoSe_2_	MoSe_2_ [102]	Pyrolysis [102]	
SnS	SnS [103]	Pyrolysis [103]	
Zn–Fe–S	Zn–Fe–S [104]	Pyrolysis [104]	
**Insertion/de-insertion materials**			
TiO_2_, Li_4_Ti_5_O_12_	TiO_2_ [105,106,107], TiO_2_–C [108], Li_4_Ti_5_O_12_ [109]	Ripening [105,106,107], Pyrolysis [109]	extreme safety/low capacity
